# Magnitude of Morning Surge in Blood Pressure Is Associated with Sympathetic but Not Cardiac Baroreflex Sensitivity

**DOI:** 10.3389/fnins.2016.00412

**Published:** 2016-09-08

**Authors:** Aaron W. Johnson, Sarah L. Hissen, Vaughan G. Macefield, Rachael Brown, Chloe E. Taylor

**Affiliations:** ^1^School of Medicine, Western Sydney UniversitySydney, NSW, Australia; ^2^School of Science and Health, Western Sydney UniversitySydney, NSW, Australia; ^3^Neuroscience Research AustraliaSydney, NSW, Australia

**Keywords:** muscle sympathetic nerve activity, microneurography, ambulatory blood pressure, cardiovascular

## Abstract

The ability of the arterial baroreflex to regulate blood pressure may influence the magnitude of the morning surge in blood pressure (MSBP). The aim was to investigate the relationships between sympathetic and cardiac baroreflex sensitivity (BRS) and the morning surge. Twenty-four hour ambulatory blood pressure was recorded in 14 young individuals. The morning surge was defined via the pre-awakening method, which is calculated as the difference between mean blood pressure values 2 h before and 2 h after rising from sleep. The mean systolic morning surge, diastolic morning surge, and morning surge in mean arterial pressures were 15 ± 2, 13 ± 1, and 11 ± 1 mmHg, respectively. During the laboratory protocol, continuous measurements of blood pressure, heart rate, and muscle sympathetic nerve activity (MSNA) were made over a 10-min period of rest. Sympathetic BRS was quantified by plotting MSNA burst incidence against diastolic pressure (sympathetic BRS_inc_), and by plotting total MSNA against diastolic pressure (sympathetic BRS_total_). Cardiac BRS was quantified using the sequence method. The mean values for sympathetic BRS_inc_, sympathetic BRS_total_ and cardiac BRS were −1.26 ± 0.26 bursts/100 hb/mmHg, −1.60 ± 0.37 AU/beat/mmHg, and 13.1 ± 1.5 ms/mmHg respectively. Significant relationships were identified between sympathetic BRS_inc_ and the diastolic morning surge (*r* = 0.62, *p* = 0.02) and the morning surge in mean arterial pressure (*r* = 0.57, *p* = 0.03). Low sympathetic BRS was associated with a larger morning surge in mean arterial and diastolic blood pressure. Trends for relationships were identified between sympathetic BRS_total_ and the diastolic morning surge (*r* = 0.52, *p* = 0.066) and the morning surge in mean arterial pressure (*r* = 0.48, *p* = 0.095) but these did not reach significance. There were no significant relationships between cardiac BRS and the morning surge. These findings indicate that the ability of the baroreflex to buffer increases in blood pressure via reflexive changes in MSNA may play a role in determining the magnitude of the MSBP.

## Introduction

In humans, blood pressure (BP) fluctuates according to a circadian rhythm, whereby it decreases during sleep and increases in the morning (Muller et al., 1989). In line with this circadian rhythm, the onset of adverse cardiovascular and cerebrovascular events such as myocardial infarction, sudden cardiac death, and stroke occur more frequently in the hours of the morning (Willich et al., 1987; Muller et al., 1989; Argentino et al., 1990; Elliot, 1998). The increase in blood pressure observed in the first 2–3 h after waking from nocturnal sleep is known as the morning surge in blood pressure (MSBP; Atkinson et al., 2014). Importantly, the MSBP has been shown to have prognostic value, as individuals with an accentuated MSBP are more likely to experience deleterious cardiovascular and cerebrovascular events (Kario et al., 2003; Li et al., 2010; Pierdomenico et al., 2014). In addition to an unfavorable prognosis, an accentuated MSBP is associated with left ventricular hypertrophy (Kaneda et al., 2005; Yano et al., 2009), greater internal carotid intima-media thickness (Yano et al., 2009), an unfavorable atherosclerotic plaque phenotype (Marfella et al., 2007) and a prothrombotic environment (Kario et al., 2011).

Previous research indicates that the sympathetic nervous system has a significant influence on the MSBP. For example, the MSBP is reduced in response to α1-adrenergic antagonist therapy (Kario et al., 2004) and centrally acting sympatholytic agents (Hashimoto et al., 2003). Furthermore, individuals with an exaggerated MSBP have higher levels of urinary catecholamine excretion (Marfella et al., 2005) and an MSBP with a larger α-adrenergic component is more intimately associated with silent cerebral infarcts (Kario et al., 2004). Investigating the relationship between muscle sympathetic nerve activity (MSNA) and the MSBP directly, it has been found that the resting levels of MSNA do not correlate to a greater MSBP (Hering et al., 2001). Whilst these findings suggest that baseline sympathetic nerve activity does not influence the magnitude of the MSBP, the mechanisms involved in blood pressure regulation are dynamic and so resting MSNA is not the only influence that the sympathetic nervous system can have on the MSBP.

The arterial baroreflex buffers spontaneous fluctutations in blood pressure through its actions on the heart and vasomotor drive to resistance vessels in, for example, skeletal muscle (Ackermann, 2004; Wehrwein and Joyner, 2013). The ability to alter heart rate or MSNA in response to a given change in arterial pressure is known as baroreflex sensitivity (BRS; Wehrwein and Joyner, 2013). BRS is clinically relevant: it plays a role in a number of diseases and is associated with a poorer prognostic outcome. For example, studies have shown that BRS is reduced in aging, diabetes, obesity, coronary artery disease, hypertension, heart failure and obstructive sleep apnoea (Skrapari et al., 2006; Monahan, 2007). An impaired sensitivity of the vascular sympathetic baroreflex (baroreflex modulation of MSNA) or cardiac baroreflex (baroreflex modulation of heart rate) could prevent the buffering of blood pressure increases that occur upon awakening, contributing to an exaggerated MSBP. Two previous studies have investigated aspects of this relationship. Okada et al. (2013) discovered that sympathetic BRS is reduced in elderly, hypertensive individuals with an exaggerated systolic MSBP, defined using the “sleep-trough” method in which morning blood pressures are compared to the trough in blood pressure during sleep (Xie et al., 2015). This method is influenced by dipping status; that is, the extent to which blood pressure decreases during sleep (Yano and Kario, 2012). It may be argued that other measures of the MSBP that are centered around the waking event may be a more realistic measure of the MSBP. Lambert et al. (2014) used a measure of morning blood pressure, known as BP_Power_, to examine the relationships between BRS, the MSBP and sympathoexcitation during a cold pressor test. The BP_Power_ approach is unique as it incorporates the rate of rise of morning BP in addition to the amplitude of the MSBP (Head et al., 2010). Lambert et al. (2014) did not find a relationship between sympathetic BRS and BP_Power_ or rate of rise, although changes in MSNA burst amplitude during the cold pressor test were positively associated with these measures of the morning surge. Despite its advantages, this novel approach to assessing the MSBP is not yet widely used thus limiting the scope for comparisons with previous studies of different populations. Furthermore, the sample of participants included individuals being treated for hypertension and a wide age range.

The major issue with research surrounding the MSBP is that it can be quantified using various methods (Stergiou et al., 2008; Atkinson et al., 2014), and the different methods used by the aforementioned studies impairs their comparability. Perhaps the most widely used method for quantifying the MSBP is the pre-awakening method. For this method, morning blood pressures are typically compared to those in the 2 or 3-h period prior to waking (Xie et al., 2015) and thus the measure is centered around waking. The aim of this paper is to investigate the relationship between BRS and the MSBP using both the pre-awakening and sleep-trough methods for quantifying the morning surge. In addition, sympathetic BRS will be quantified using both “burst incidence” (threshold technique) and “total MSNA” methods in order to account for baroreflex-driven changes in MSNA burst amplitude. It is hypothesized that individuals with reduced sympathetic BRS have larger pre-awakening morning surges in blood pressure.

## Materials and methods

### Participants and ethics

This study was conducted under the approval of the Human Research Ethics Committee of Western Sydney University and satisfied the Declaration of Helsinki. Fourteen healthy young volunteers (12 male, 2 female; age 19–27 years) participated in this study. All participants provided written informed consent prior to taking part. Participants who had known cardiovascular, neuromuscular, or respiratory conditions, as well as those who smoked or took regular medication were excluded. The changes in hormone levels associated with the menstrual cycle have been proven to influence MSNA and sympathetic BRS (Minson et al., 2000). Accordingly, females participated in all experimental protocols during the early follicular (low-hormone) phase of their menstrual cycle.

### Experimental design and measurements

#### Ambulatory protocol

Ambulatory blood pressure was recorded using an ambulatory blood pressure monitor (Spacelabs 90217A, Spacelabs Healthcare, Snoqualmie WA, USA) for 24 h, to ensure that recordings were taken during periods of both sleep and wake. The monitors were programmed to take blood pressure readings every 20 min during wakefulness and every 30 min during sleep over the 24 h. Before the recording, participants indicated the times that they normally slept and the monitor was set accordingly to minimize disturbance to sleep. During the period of recording, participants were required to complete an activity diary indicating activities that were undertaken during the day as well as their sleep and wake times. Actigraphy was completed simultaneously using an activity monitor (Actigraph wGT3X-BT, Actigraph, Pensacola, FL) worn on the waist to confirm the time of waking. Participants were instructed to undertake their usual activities but to refrain from strenuous exercise and any activities that were not normally a part of their daily routine. On completion of the recording, participants were asked to indicate the quality of their sleep.

#### Laboratory protocol

At a time separate from the period of ambulatory blood pressure recordings, participants attended the Human Autonomic Laboratory at the School of Medicine, WSU (Campbelltown, NSW, Australia). All experiments took place in the morning, beginning at 0800h. The following variables were recorded non-invasively: heart rate (ECG), blood pressure (Finometer Pro, Finapres Medical Systems, Amsterdam, The Netherlands), and respiration (Pneumotrace, UFI, Morro Bay CA, USA). MSNA was recorded from fascicles of the common peroneal nerve supplying the pretibial flexors via tungsten microelectrodes (FHC, Bowdoinham, ME, USA) inserted percutaneously at the level of the fibular head. Multi-unit neural activity was amplified (gain 20,000, bandpass 0.3–5.0 kHz) using an isolated amplifier (NeuroAmp EX, ADInstruments, Sydney, Australia) and stored on computer (10 kHz sampling) using a computer-based data acquisition and analysis system (PowerLab 16SP hardware and LabChart 6 Pro software; ADInstruments, Sydney, Australia). Mean voltage of the nerve signal was computed via the root mean square (RMS) feature in LabChart, using a moving average of 200 ms. Participants were seated in a semi reclined posture in a comfortable chair with the legs supported in the extended position. The variables described above were then continuously measured over a 10-min period.

### Data analysis

#### Ambulatory blood pressure characteristics and MSBP

**Figure 1:**
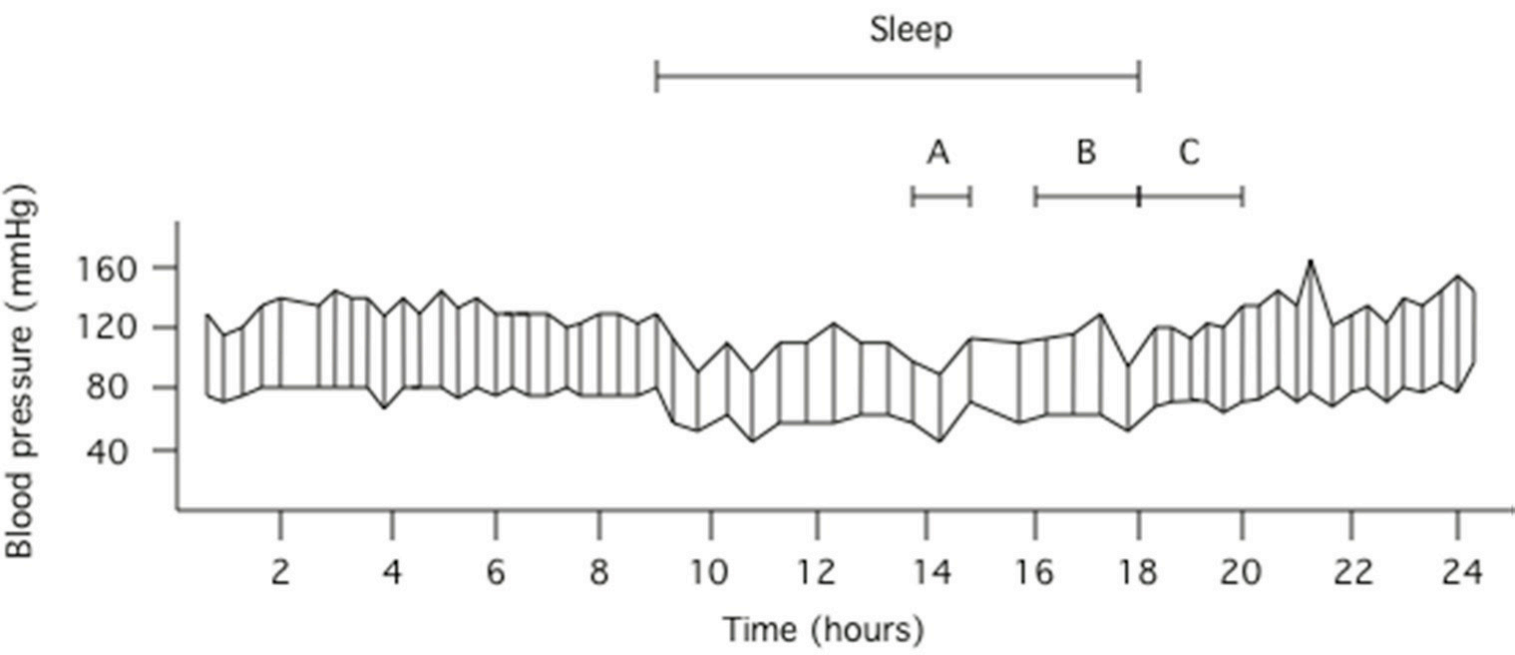
**Ambulatory blood pressure data obtained in a 22 year old male**. The top line respresents fluctuations in systolic blood pressure whilst the bottom line represents fluctations in diastolic blood pressure during the 24 h recording. Using systolic blood pressure as the example, interval A represents the three systolic blood pressure values centered around the lowest systolic blood pressure obtained during sleep, interval B represents the 2 h prior to rising from sleep and interval C represents the 2 h after rising from sleep. Pre-awakening morning surge in blood pressure is determined via the difference between the mean of the systolic blood pressures captured during interval C and the mean of the systolic blood pressures captured during interval B. Sleep-trough morning surge in blood pressure is determined via the difference between the mean of the systolic blood pressures captured during interval C and the mean of the three systolic blood pressures represented by interval A.

For each participant, mean values of systolic BP, diastolic BP, MAP, and heart rate were calculated for each time period of the day (across the entire 24 h, during times of wakefulness and during times of sleep). Two widely recognized definitions of the MSBP were quantified for each participant: (i) the pre-awakening morning surge and (ii) the sleep-trough morning surge (Xie et al., 2015). The *pre-awakening morning surge* was determined via the difference between the average BP recordings 2 h after rising from sleep and the average BP recordings over 2 h before rising from sleep (Figure 1). The *sleep-trough morning surge* was determined as the difference between the average BP over 2 h after rising from sleep and the average of three consecutive BP recordings, centered on the lowest reading during sleep (Figure 1). Both definitions of MSBP were quantified using systolic BP, diastolic BP, and MAP.

#### Laboratory data analysis

**Figure 2:**
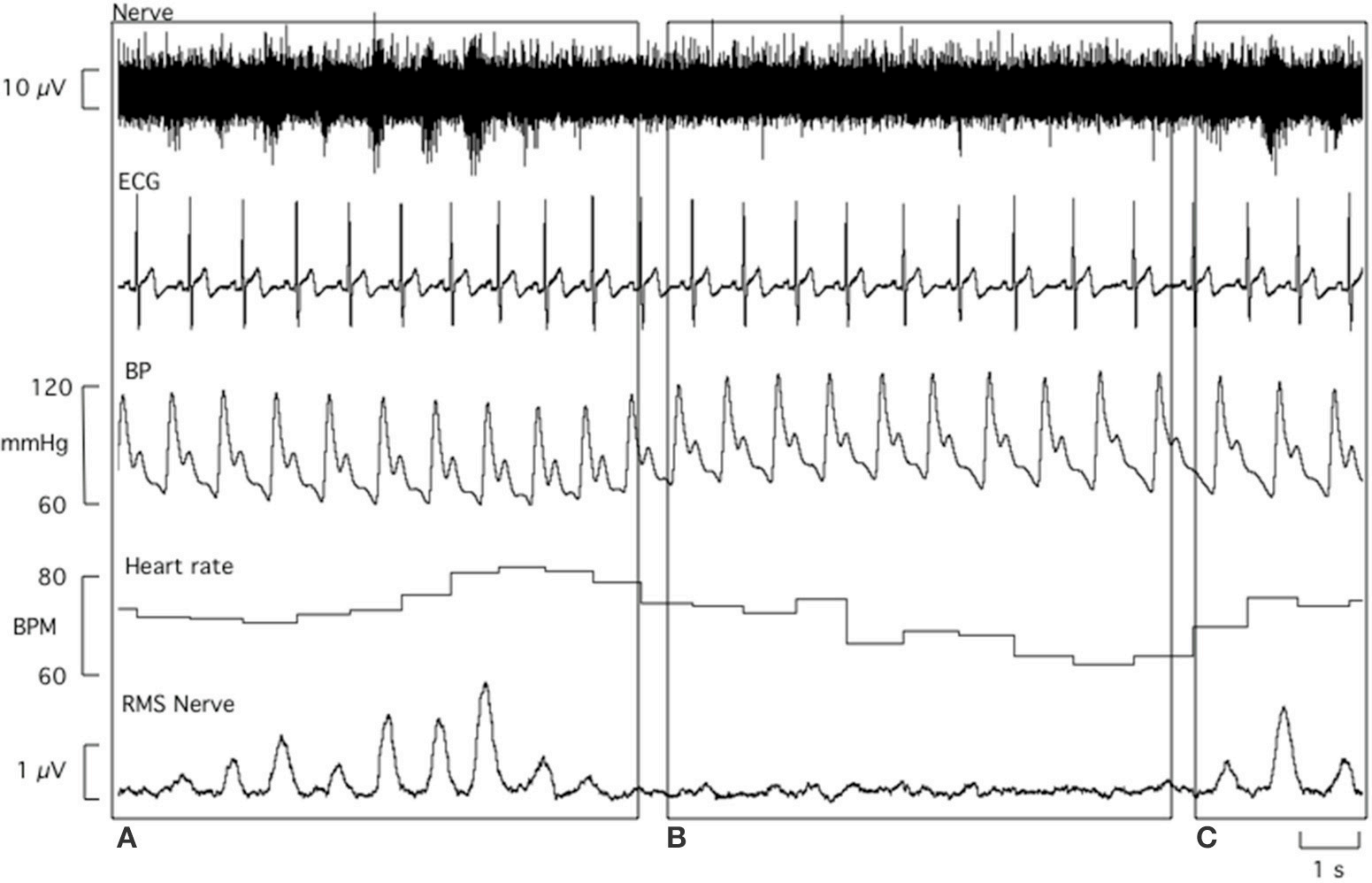
**Laboratory data obtained from a 21 year old male**. The baroreflex coordinates an increase in both MSNA burst incidence and heart rate in response to a transient decrease in blood pressure **(A)**. Conversely, rising systolic and diastolic blood pressure causes a fall in heart rate and inhibition of MSNA bursts **(B)**. MSNA burst incidence increases again in response to a drop in diastolic blood pressure **(C)**.

All laboratory data analysis was performed by one investigator to minimize any subjective differences in defining sympathetic bursts. Beat-to-beat values were extracted from LabChart (ADI Instruments, Sydney, Australia) for systolic BP, diastolic BP, R-R interval, and MSNA. The experimental records for one participant are shown in Figure 2. MSNA bursts were detected and measured from the RMS nerve signal using a custom-written analysis program (LabView, National Instruments, USA). For each participant, the number of MSNA bursts per 100 heart beats (MSNA burst incidence) and number of MSNA bursts per minute (MSNA burst frequency) were calculated. Sympathetic BRS and cardiac BRS analyses were performed using the spontaneous techniques described below.

#### Sympathetic baroreflex sensitivity: burst incidence method

Sympathetic BRS was quantified by plotting burst incidence against diastolic blood pressure, as described by Kienbaum et al. (2001). Prior to the determination of sympathetic BRS, the nerve trace was shifted to account for the sympathetic baroreflex conduction delay and this was adjusted for each participant to account for inter-individual differences in burst latency. The mean shift applied was 1.31 ± 0.03 s. To help remove non-baroreflex stimuli, the diastolic pressure values of each cardiac cycle were assigned to 3 mmHg bins for each participant (Ebert and Cowley, 1992; Taylor et al., 2011). The corresponding MSNA burst incidence (bursts/100 HB) was then calculated for each bin. Sympathetic BRS was quantified by plotting MSNA burst incidence against the mean diastolic BP for each bin. A weighting procedure was applied according to the number of cardiac cycles for each diastolic BP bin, as the highest and lowest diastolic pressures contain fewer cardiac cycles (Kienbaum et al., 2001). The acceptance level for the linear regression analyses was set at *r* > 0.5 (Rudas et al., 1999; Hart et al., 2011b; Okada et al., 2013). These baroreflex values will be referred to as sympathetic BRS_inc_ for the purpose of differentiating it from other methods of obtaining sympathetic BRS.

#### Sympathetic baroreflex sensitivity: total MSNA method

All sympathetic bursts obtained during the MSNA recording were normalized to the largest burst, with the largest being assigned a total integrated nerve activity value of 1000. The 0 nerve activity value was determined from the mean voltage during a >5 s period of neural silence between sympathetic bursts. Diastolic blood pressures for each cardiac cycle were assigned to 3 mmHg bins. Total MSNA was determined for each bin using the segregated signal averaging approach described by Halliwill (2000). In this approach, 2-s windows synchronized with the R wave were used to determine the MSNA for the cardiac cycles within each 3 mmHg diastolic pressure bin. MSNA windows were time shifted so as to account for the latency between R waves and sympathetic bursts and the time shift varied from individual to individual. The mean time shift was 1.33 ± 0.04 s. The total integrated nerve activity was determined using the area under the signal-averaged curve for each bin and expressed as arbitrary units (AU) per beat. Linear regression was performed by plotting total MSNA against diastolic BP with the acceptance level set at *r* > 0.5 (Rudas et al., 1999; Hart et al., 2011b; Okada et al., 2013). Data points were weighted according to the number of cardiac cycles per diastolic BP bin. These sympathetic BRS values will be referred to as “sympathetic BRS_total_” to differentiate these values from the sympathetic BRS values obtained via the burst incidence method.

#### Cardiac baroreflex sensitivity: sequence method

The sequence method was used to determine cardiac BRS. In this method, up and down sequences are identified. “Up sequences” consist of three or more consecutive cardiac cycles for which there is a rise in systolic BP coinciding with lengthening of the R-R interval. “Down sequences” consist of three or more cardiac cycles for which there is a fall in systolic BP coinciding with shortening of the R-R interval. The threshold for changes in systolic BP was set at 1 mmHg and the threshold for changes in R-R interval was set at 6 ms (Parati et al., 1988). Sequences containing changes smaller than these thresholds were not used in the assessment of cardiac BRS. To account for baroreflex delays, systolic BP values were matched to either the concurrent heartbeat for R-R intervals of 800 ms or a 1 beat delay for shorter heart periods, typically 500–800 ms (Eckberg and Eckberg, 1982). Cardiac BRS was quantified by plotting R-R interval against systolic BP for each sequence (*r* ≥ 0.8 acceptance level) and taking the average for up and down sequences combined (Monahan et al., 2001; Okada et al., 2013). Values of cardiac BRS were accepted when the number of sequences was ≥3 for both up and down sequences.

### Statistical analysis

In order to test the hypothesis that the morning surge in BP is negatively correlated to BRS, separate linear regression analyses were performed to determine the relationships between MSBP and BRS variables. The analyses were performed for systolic, diastolic and MAP components of MSBP, relating each to sympathetic BRS and cardiac BRS. Linear regression analyses were also performed to examine the relationships between BRS variables and secondary ambulatory blood pressure characteristics (mean 24 h BP, BP whilst awake, and BP whilst asleep). All statistical analyses were performed using Prism 6 (GraphPad, USA) and the alpha level was set at 0.05.

## Results

### Participant characteristics

Recordings of MSNA were successfully obtained in all 14 participants. The mean age of these participants was 23 ± 1 years and mean body mass index was 24.3 ± 0.8 kg/m^2^. Resting MSNA burst incidence and MSNA burst frequency were 44 ± 5 bursts/100 HB and 30 ± 3 bursts/min, respectively. Neither MSNA burst frequency nor MSNA burst incidence was related to the measures of the MSBP (*P* > 0.05).

### Ambulatory blood pressure characteristics and MSBP

At least two thirds of the blood pressure readings were successful for all participants, thus meeting the minimum requirements for ambulatory blood pressure recordings (O'Brien et al., 2005). None of the participants reported severely disrupted sleep. The mean number of blood pressure recordings over the 24 h period, during sleep and during wake were 61 ± 1 (range 51–67), 16 ± 1 (range 12–22), and 44 ± 1 (range 37–50), respectively. The average time of going to sleep was 23:43 h (range 22:22–00:44 h) and the average time of rising was 07:28 h (range 05:10–10:12 h). The mean hours of sleep was 8.2 ± 2.2 h. Mean values for systolic BP, diastolic BP, MAP, and heart rate over 24 h and during sleep and times of wakefulness are displayed in Table 1. All cardiovascular variables were significantly lower during sleep than wakefulness (*p* < 0.05). The mean MSBP values are displayed in Table 2. Linear regression analyses revealed no significant relationships between BRS (sympathetic BRS_inc_, sympathetic BRS_total_, cardiac BRS) and blood pressure averaged over the 24-h recording period, wakefulness or sleep (*p* > 0.05).

**Table 1:** **Mean values for systolic BP, diastolic BP, MAP and heart rate over 24 h, and during wakefulness and sleep (***n*** = 14)**.

**Cardiovascular variable**	**24-h**	**Wakefulness**	**Sleep**
Systolic BP (mmHg)	120 ± 3	126 ± 3	112 ± 3^*^
Diastolic BP (mmHg)	69 ± 1	74 ± 1	62 ± 2^*^
MAP (mmHg)	86 ± 1	90 ± 1	80 ± 2^*^
Heart rate (bpm)	69 ± 2	72 ± 2	59 ± 2^*^

BP, blood pressure; MAP, mean arterial pressure.

* p < 0.05 vs. wakefulness.

**Table 2:** **Mean MSBP values (***n*** = 14)**.

**MSBP definition**	**Systolic MSBP (mmHg)**	**Diastolic MSBP (mmHg)**	**Morning surge in MAP (mmHg)**
Pre-awakening	15 ± 2	13 ± 1	11 ± 1
Sleep-trough	21 ± 2	18 ± 2	16 ± 2

MSBP, morning surge in blood pressure; MAP, mean arterial pressure.

### Baroreflex sensitivity

#### Sympathetic baroreflex sensitivity: burst incidence method

Significant sympathetic BRS_inc_ slopes (*r* > 0.5) were obtained for all 14 participants. The mean sympathetic BRS_inc_ was −1.26 ± 0.26 bursts/100 hb/mmHg. An outlier with a sympathetic BRS_inc_ value of −3.9 bursts/100 hb/mmHg was identified (>1.5 interquartile ranges below first quartile).

#### Sympathetic baroreflex sensitivity: total MSNA method

Thirteen participants exhibited significant sympathetic BRS_total_ slopes (*r* > 0.5). The participant that did not was excluded from analyses involving sympathetic BRS_total_. The mean sympathetic BRS_total_ was −1.60 ± 0.37 AU/beat/mmHg. The BRS_total_ value for the same participant was identified as an outlier (−5.4 AU/beat/mmHg).

#### Cardiac baroreflex sensitivity: sequence method

All 14 participants had a sufficient number of up and down sequences to allow determination of cardiac BRS. The mean slope obtained for cardiac BRS was 13.1 ± 1.5 ms/mmHg.

### Relationship between baroreflex sensitivity and MSBP

#### Sympathetic BRS_inc_ and “pre-awakening” MSBP

**Figure 3:**
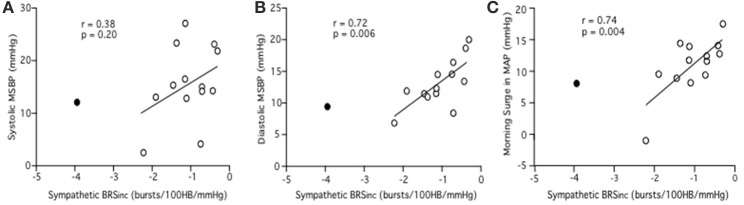
**Relationships between sympathetic BRS_**inc**_ and the systolic (A), diastolic (B) and mean arterial (C) components of the morning surge in blood pressure as determined via the pre-awakening method.** Closed circle = outlier.

Linear regression analysis did not reveal a significant relationship between sympathetic BRS_inc_ and pre-awakening systolic MSBP (*r* = 0.33, *p* = 0.25). However, a significant negative relationship was identified between sympathetic BRS_inc_ and pre-awakening diastolic MSBP (*r* = 0.62, *p* = 0.02) and between sympathetic BRS_inc_ and pre-awakening morning surge in MAP (*r* = 0.57, *p* = 0.03). Since sympathetic BRS values are negative, a positive *r*-value indicates that low sympathetic BRS is associated with a larger MSBP. With the outlier removed the findings were consistent albeit with steeper linear regression slopes. The relationship remained significant between sympathetic BRS_inc_ and pre-awakening diastolic MSBP (*r* = 0.72, *p* = 0.006; Figure 3B) and between sympathetic BRS_inc_ and pre-awakening morning surge in MAP (*r* = 0.74, *p* = 0.004; Figure 3C). There was still no significant relationship between sympathetic BRS_inc_ and pre-awakening systolic MSBP (*r* = 0.38, *p* = 0.20; Figure 3A).

#### Sympathetic BRS_inc_ and “sleep-trough” MSBP

Linear regression analyses did not reveal any significant relationships between sympathetic BRS_inc_ and sleep-trough systolic MSBP (*r* = 0.19, *p* = 0.52), sleep-trough diastolic MSBP (*r* = 0.34, *p* = 0.24) or sleep-trough morning surge in MAP (*r* = 0.35, *p* = 0.23). Removal of the outlier revealed a negative relationship between sympathetic BRS_inc_ and sleep-trough morning surge in MAP (*r* = 0.55, *p* = 0.05). The relationships between sympathetic BRS_inc_ and sleep-trough systolic MSBP (*r* = 0.38, *p* = 0.20) and sleep-trough diastolic MSBP (*r* = 0.45, *p* = 0.12) remained insignificant with removal of the outlier.

#### Sympathetic BRS_total_ and “pre-awakening” MSBP

**Figure 4:**
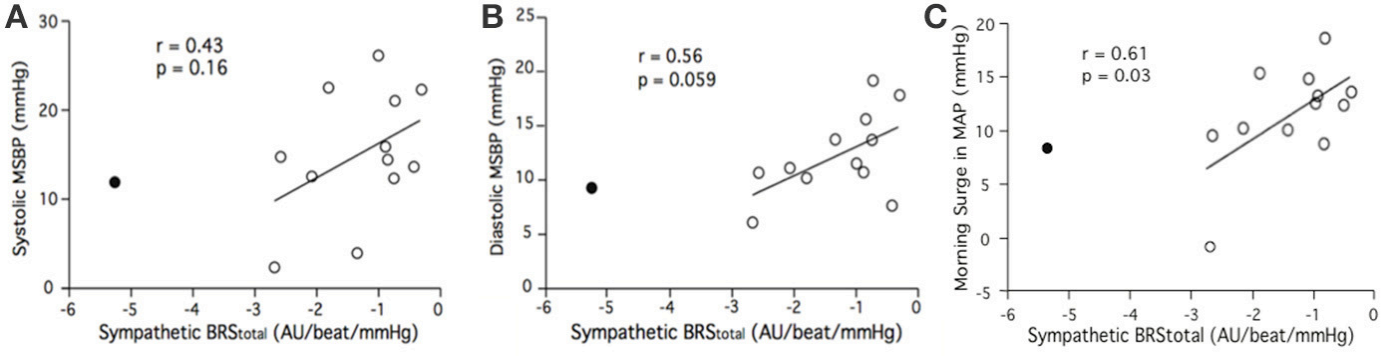
**Relationships between sympathetic BRS_**total**_ and the systolic (A), diastolic (B) and mean arterial (C) components of the morning surge in blood pressure (MSBP) as determined via the pre-awakening method.** Closed circle = outlier.

Linear regression did not reveal a significant relationship between sympathetic BRS_total_ and pre-awakening systolic MSBP (*r* = 0.36, *p* = 0.23). There was a relationship between sympathetic BRS_total_ and pre-awakening diastolic MSBP but this did not reach statistical significance (*r* = 0.52, *p* = 0.066). There was also a trend for a relationship between sympathetic BRS_total_ and pre-awakening morning surge in MAP (*r* = 0.48, *p* = 0.095). This trend was significant with removal of the outlier noted above (*r* = 0.61, *p* = 0.03; Figure 4C). With the removal of the outlier the relationship between sympathetic BRS_total_ and pre-awakening morning surge in DBP neared significance (*r* = 0.56, *p* = 0.059; Figure 4B). There was still no significant relationship between sympathetic BRS_total_ and pre-awakening systolic MSBP (*r* = 0.43, *p* = 0.16; Figure 4A).

#### Sympathetic BRS_total_ and “sleep-trough” MSBP

Linear regression analyses revealed no significant relationships between sympathetic BRS_*total*_ and sleep-trough systolic MSBP (*r* = 0.25, *p* = 0.41), sleep-trough diastolic MSBP (*r* = 0.46, *p* = 0.11) or sleep-trough morning surge in MAP (*r* = 0.40, *p* = 0.18). Removal of the outlier revealed a trend for relationship between sympathetic BRS_total_ and sleep-trough morning systolic MSBP that did not reach significance (*r* = 0.52, *p* = 0.08). With the outlier removed, there were significant relationships between sympathetic BRS_total_ and sleep-trough diastolic MSBP (*r* = 0.67, *p* = 0.02) and sleep-trough morning surge in MAP (*r* = 0.66, *p* = 0.02).

#### Cardiac baroreflex sensitivity and “pre-awakening” MSBP

**Figure 5:**
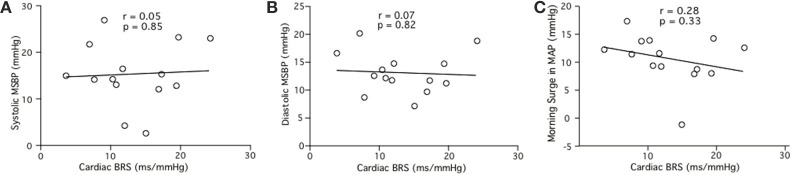
**Relationships between cardiac BRS and the systolic (A), diastolic (B) and mean arterial (C) components of the morning surge in blood pressure (MSBP) as determined via the pre-awakening method**.

Linear regression analysis did not reveal significant relationships between cardiac BRS and MSBP for pre-awakening systolic MSBP (*r* = 0.05, *p* = 0.85; Figure 5A), pre-awakening diastolic MSBP (*r* = 0.07, *p* = 0.82; Figure 5B) or pre-awakening morning surge in MAP (*r* = 0.28, *p* = 0.15; Figure 5C).

#### Cardiac baroreflex sensitivity and “sleep-trough” MSBP

Linear regression did not reveal significant relationships between cardiac BRS and sleep-trough systolic MSBP (*r* = 0.06, *p* = 0.85), sleep-trough diastolic MSBP (*r* = 0.12, *p* = 0.68) or sleep-trough morning surge in MAP (*r* = 0.15, *p* = 0.44).

## Discussion

The aim of this study was to determine the relationship between BRS and the MSBP in healthy, young individuals. Our major finding is that the magnitude of the pre-awakening morning surge in diastolic and mean arterial pressure is greater in individuals who have a lower sympathetic but not cardiac BRS. This supports the hypothesis that the ability of the sympathetic baroreflex to buffer increases in blood pressure may play a role in determining the magnitude of the morning surge.

### Sympathetic baroreflex sensitivity and the MSBP

In this study, the magnitudes of the pre-awakening morning surge in diastolic and mean arterial pressure were exaggerated in individuals with lower sympathetic BRS_inc_. By contrast, no significant relationships were found between pre-awakening systolic MSBP and sympathetic BRS. Similar trends were found between sleep-trough MSBP variables and sympathetic BRS but none reached significance, except with the removal of an outlier. It should be noted, however, that the outlier in the present data set (>1.5 interquartile ranges below the first quartile) still represents a typical sympathetic BRS value for a healthy young as reported by our research group (Taylor et al., 2015) and others (Hart et al., 2011b). The “sleep-trough” method is more likely to be influenced by dipping status, as it is centered around the lowest BP reading obtained during sleep. “Dipping status” is also a risk factor for cardiovascular and cerebrovascular disease (Kario et al., 2001; Yano and Kario, 2012), which may confound the prognostic significance of the MSBP when using the sleep-trough method. The pre-awakening MSBP is centered around the waking event and this could explain why pre-awakening MSBP was more intimately related to sympathetic BRS. However, Okada et al. (2013) examined the sleep-trough systolic MSBP and found that it is associated with sympathetic BRS_inc_, at least in elderly, hypertensive individuals. This relationship was not present in normotensive individuals, although the morning surge in diastolic and MAP were not examined.

In the current study, pre-awakening diastolic MSBP and morning surge in MAP were significantly correlated to sympathetic BRS, whilst systolic MSBP was not. The occurrence of bursts of MSNA is driven by physiological fluctuations in diastolic BP (Sundlof and Wallin, 1987) and, as a result, diastolic BP is more intimately related to sympathetic baroreflex function. Considering this, it seems logical that the diastolic MSBP, as opposed to the systolic MSBP, is correlated with sympathetic BRS. Previous research concerning the risks associated with the MSBP tends to have involved the use of systolic BP to quantify the morning surge (Xie et al., 2015). As a result, the elevated cardiovascular risk associated with the systolic MSBP has been well established. Although the prognostic significance of the diastolic MSBP is less studied, research suggests that an exaggerated diastolic MSBP may be associated with increased risk of cardiovascular events. In a large study by Li et al. (2010), involving 5645 participants, an increased hazard ratio was identified for all-cause mortality with both an exaggerated pre-awakening systolic and diastolic MSBP. An exaggerated pre-awakening diastolic MSBP (≥21.6 mmHg), was associated with a hazard ratio of 1.26 (CI 1.01–1.56) for all cause mortality and 1.40 (CI 1.00–1.96) for fatal and non-fatal cardiac events. For all cardiovascular and cerebrovascular events, a hazard ratio of 1.21 (CI 0.95–1.54) was determined, although this did not reach statistical significance. Nevertheless, these findings suggest that the pre-awakening diastolic MSBP may still have some predictive value. Further research should be conducted to confirm the prognostic significance of diastolic MSBP.

### Cardiac BRS and the MSBP

We have previously identified a significant but modest relationship between cardiac and sympathetic BRS in young individuals (Taylor et al., 2015). The findings suggested that cardiac BRS predicts only around 10% of the variability in sympathetic BRS and therefore one cannot reliably predict the other. In support of the current study, it also suggests that a relationship may exist between the morning surge and cardiac or sympathetic BRS but not necessarily both. In contrast to sympathetic BRS, which is determined using diastolic BP, cardiac BRS is quantified using systolic BP (Parati et al., 1988). Therefore, similar to the relationship between sympathetic BRS and diastolic MSBP, it may be speculated that cardiac BRS is associated with an exaggerated systolic MSBP. However, we did not find a relationship between cardiac BRS and either the systolic or the diastolic component of the MSBP. Cardiac BRS may be unrelated to the MSBP due to the fact that it represents a marker of reflex vagal modulation. Our findings support the studies by Okada et al. (2013) and Lambert et al. (2014) in which no significant relationship was reported between cardiac BRS and either the morning surge in systolic pressure or MAP, respectively. Our previous work indicates that when males and females are assessed separately the relationship between cardiac and sympathetic BRS is significant only for females. In light of this, the study of larger groups of males and females is warranted to determine whether the present findings are influenced by gender. Since the control of blood pressure via sympathetic vasoconstriction appears to be more dominant in males than females (Hart et al., 2011a, 2014), it may be postulated that the sympathetic baroreflex has a greater influence on the morning surge in males than in females.

### Methodological considerations

A major issue with the MSBP is that there has been no consensus regarding the precise definition of the MSBP and as a consequence, researchers have utilised a number of different methods to quantify the morning surge (Stergiou et al., 2008; Atkinson et al., 2014). For the reasons described earlier, the pre-awakening definition may be considered a more realistic measure of the MSBP. However, some participants had morning blood pressure increases that spanned more than 2 h after rising and this is a limitation of the method used. Nonetheless, the risk associated with the pre-awakening morning surge has been more extensively studied (Xie et al., 2015) than definitions that include BP readings over a longer time period after rising, which is why these definitions were not considered in the present study. The BP_Power_ is a promising method as it provides a comprehensive evaluation of an individual's MSBP. It has also been found that the rate of rise of the MSBP is greater in hypertensives (Head et al., 2006) and is an independent risk factor for stroke and myocardial infarction (Luo et al., 2013). Further study of this method in the context of sympathoexcitation and circadian variation may allow the mechanisms involved in the MSBP to be unraveled. Nevertheless, the fact that we report significant correlations between the MSBP and sympathetic BRS using the pre-awakening method obviate the use of the BP_Power_ method in the current study.

To our knowledge, only two studies have previously examined the relationship between sympathetic BRS and the MSBP (Okada et al., 2013; Lambert et al., 2014) with both studies using the burst incidence method to quantify sympathetic BRS. The burst incidence method, otherwise known as the threshold technique, involves plotting MSNA burst incidence against diastolic BP and is associated with high success rates for significant baroreflex slopes (Kienbaum et al., 2001). It is important to recognize that the burst incidence method does not incorporate changes in MSNA burst amplitude. The present study is the first to examine the relationship between sympathetic BRS_total_ and the MSBP, thus allowing the incidence and the amplitude of MSNA bursts to be taken into account. The total MSNA method (Halliwill, 2000) involves plotting total MSNA against diastolic blood pressure in order to quantify sympathetic BRS and incorporates both burst strength and burst incidence. Whilst we were unable to obtain a significant BRS_total_ slope in one participant, the success rate (13/14, 93%) remained comparable with that of the burst incidence method (14/14, 100%). It is interesting to note that when an outlier was removed, sympathetic BRS_inc_ and sympathetic BRS_total_ were both correlated with the MSBP. This may be expected because the two methods are not mutually exclusive. However, it tends to suggest that the ability of the baroreflex to modulate the occurrence of bursts (burst incidence) may be the more influential factor in determining the magnitude of the MSBP, as the addition of burst strength into the analyses had little effect on the relationships between sympathetic BRS and MSBP variables. This supports the concept that changes in MSNA burst incidence are driven primarily by the baroreflex whereas the modulation of MSNA burst amplitude may involve the influence of other modulators, such as chemoreceptors (Malpas et al., 1996).

This study provides evidence to elucidate the influence of the sympathetic nervous system on the MSBP. Although the study of healthy, young individuals limits the ability to generalize the results to clinical populations, it is known that hypertension is associated with both exaggerated MSBP (Kario et al., 2003) and diminished cardiac BRS (Grassi et al., 1998). Further examination of sympathetic baroreflex function and its relation to the pre-awakening MSBP in this population is warranted. However, the current findings suggest there is potential for the use of appropriately timed antihypertensive therapy, specifically targeting the sympathetic nervous system, that may be useful in reducing the cardiovascular morbidity and mortality in individuals with an exaggerated MSBP.

### Limitations

We note that the present study is limited by a relatively small sample size and it is possible that investigation of a larger population may have allowed further relationships to be identified. However, the study was sufficiently powered to detect a significant relationship between the diastolic and mean arterial components of the pre-awakening MSBP and sympathetic BRS. This is a novel finding in healthy, young populations. Although the baroreflex response to rising pressures may be considered most relevant to the MSBP, we did not perform separate BRS analyses with respect to rising and falling pressures. The sequence method lends itself to quantifying cardiac BRS in response to rising and falling pressures, but the assessment of sympathetic BRS does not involve the detection of sequences and therefore does not allow for such differentiation. Hart et al. (2011b) have developed a sequence technique for sympathetic BRS, but this comes with its own limitations as it involves sequences of only two consecutive cardiac cycles and does not provide significant slopes in all individuals. For these reasons separate analyses were not performed for rising and falling pressures for sympathetic BRS, and rising and falling cardiac BRS values were pooled for consistency. The technique of ambulatory blood pressure monitoring is also associated with some limitations. Given the differing schedules of participants during the day, the precise times of wake and sleep for each participant were unable to be controlled. However, individuals who took part in shift work or who had abberant sleep patterns were excluded from the study. It is possible that BRS changes under different conditions and throughout the day. Whilst all recordings were made at rest, our findings are based upon measurements taken at only one time point during the day. However, we have previously shown that sympathetic BRS is not significantly different between the morning and afternoon (Hissen et al., 2015). The ambulatory and laboratory protocols were not performed on the same morning and it is acknowledged that this is a limitation and may have influenced the findings.

## Conclusion

We have uncovered significant relationships between sympathetic BRS and both the diastolic and mean arterial components of the pre-awakening MSBP in healthy, young individuals; the higher the MSBP, the lower the sympathetic BRS. This finding suggests that the ability of the baroreflex to buffer increases in blood pressure may have an influence on the magnitude of the MSBP.

## Author contributions

Experiments were performed in the School of Medicine (Western Sydney University). All authors were involved in the design of the experiments and/or data acquisition and analysis of the data, as well as the writing or editing of this manuscript. All authors approved the final version of the manuscript and agree to be accountable for all aspects of the work.

### Conflict of interest statement

The authors declare that the research was conducted in the absence of any commercial or financial relationships that could be construed as a potential conflict of interest.
